# Role of Galectins in the Liver Diseases: A Systematic Review and Meta-Analysis

**DOI:** 10.3389/fmed.2021.744518

**Published:** 2021-10-27

**Authors:** Yang An, Shixue Xu, Yiting Liu, Xiangbo Xu, Cyriac Abby Philips, Jiang Chen, Nahum Méndez-Sánchez, Xiaozhong Guo, Xingshun Qi

**Affiliations:** ^1^Meta-Analysis Study Group, Department of Gastroenterology, General Hospital of Northern Theater Command, Shenyang, China; ^2^Postgraduate College, Shenyang Pharmaceutical University, Shenyang, China; ^3^Department of Physical Examination Center, The First Affiliated Hospital, China Medical University, Shenyang, China; ^4^The Liver Unit and Monarch Liver Laboratory, Cochin Gastroenterology Group, Ernakulam Medical Center, Kochi, India; ^5^Liver Research Unit Medica Sur Clinic and Foundation and Faculty of Medicine, National Autonomous University of Mexico, Mexico City, Mexico

**Keywords:** galectins, hepatocellular carcinoma, cirrhosis, hepatitis, fibrosis

## Abstract

**Background:** Galectins, a family of β-galactoside-binding proteins, are related to the development and progression of various human diseases such as cancer, heart failure, and chronic kidney disease. However, its role in liver diseases is unclear.

**Methods:** The PubMed, Embase, and Cochrane Library databases were searched. Hazard ratios (HRs), odds ratios (ORs), and mean differences (MDs) with 95% CIs were pooled to evaluate the association of the galectins with the outcomes and risk of liver diseases by a random effects model.

**Results:** Thirty three studies involving 43 cohorts and 4,168 patients with liver diseases were included. In the patients with hepatocellular carcinoma (HCC), high expression of galectin-1 and -3 in the tissues was significantly associated with worse overall survival (galectin-1: HR = 1.94, 95% CI = 1.61–2.34, *p* < 0.001; galectin-3: HR = 3.29, 95% CI = 1.62–6.68, *p* < 0.001) and positive vascular invasion (galectin-1: OR = 1.74, 95% CI = 1.18–2.58, *p* = 0.005; galectin-3: OR = 2.98, 95% CI = 1.58–5.60, *p* = 0.001); but, high expression of galectin-4 and −9 in the tissues was significantly associated with better overall survival (galectin-4: HR = 0.53, 95% CI = 0.36–0.79, *p* = 0.002; galectin-9: HR = 0.56, 95% CI = 0.44–0.71, *p* < 0.001) and negative vascular invasion (galectin-4: OR = 0.36, 95% CI = 0.19–0.72, *p* = 0.003; galectin-9: OR = 0.60, 95% CI = 0.37–0.97, *p* = 0.037). Serum galectin-3 level was significantly higher in HCC (MD = 3.06, 95% CI = 1.79–4.32, *p* < 0.001), liver failure (MD = 0.44, 95% CI = 0.23–0.66, *p* < 0.001), liver cirrhosis (MD = 1.83, 95% CI = 1.15–2.51, *p* < 0.001), and chronic active hepatitis B (MD = 18.95, 95% CI = 10.91–27.00, *p* < 0.001); serum galectin-9 level was significantly higher in HCC (MD = 3.74, 95% CI = 2.57–4.91, *p* < 0.001) and autoimmune hepatitis (MD = 8.80, 95% CI = 7.61–9.99, *p* < 0.001).

**Conclusion:** High galectin-1 and -3 and low galectin-4 and -9 expression indicate worse outcomes of patients with HCC. Serum galectin-3 and -9 levels are positively associated with the risk of chronic liver diseases.

## Introduction

Liver diseases, including chronic hepatitis, liver fibrosis or cirrhosis, acute liver injury or liver failure, and hepatocellular carcinoma (HCC), are a major global health burden. They are often subtle, but potentially lethal (1). According to the report of the Global Burden of Disease Study 2019, there are 79,200 deaths from acute hepatitis (2), 1,470,000 deaths from liver cirrhosis and other chronic liver diseases (3), and 485,000 deaths from HCC (4) in the world. Early assessment and identification of liver diseases by molecular biomarkers are clinically important.

Galectins are a family of lectins composed of one or two carbohydrate recognition domains (CRDs) that bind to the β-galactoside-containing glycans (5). Galectins are classified into three groups according to their molecular-structural characteristics: “prototype” galectins with a single CRD (i.e., galectin-1,-2,-5,-7,-10,-11,-13,-14,-15, and -16); “chimeric-type” galectins (i.e., galectin-3) with the tandem repeats of proline- and glycine-rich short stretches fused onto the CRD; and “tandem repeat”-type galectins with two distinct CRDs (i.e., galectin-4,-6,-8,-9, and -12) (6). Galectins are responsible for the regulation of premessenger RNA (mRNA) splicing, cell cycle, cell growth, and cell apoptosis (7), and the development and/or progression of many human diseases, including cancer, heart failure, and chronic kidney disease (8).

Galectins play a regulatory role in liver diseases by binding their CRDs to the glycoconjugates expressed in the hepatocytes (9). Abnormal expression of the galectins may be related to the development of hepatitis and liver fibrosis/cirrhosis and the progression of HCC (10). In this study, we conducted a systematic review and meta-analysis to evaluate the role of galectins in various liver diseases.

## Methods

This meta-analysis was performed in accordance with the Preferred Reporting Items for Systematic Reviews and Meta-Analyses (PRISMA) statement (11).

### Registration

The registration number was CRD42020210038 in the PROSPERO.

### Literature Search

The literature was searched via the PubMed, Embase, and Cochrane Library databases from the earliest available publication until September 18, 2020. Search items were as follows: “(galectin)” and “(liver)” or “(hepatic)” or “(hepatitis)” or “(hepatocellular)” or “(fibrosis)” or “(failure).” There was no language restriction.

### Selection Criteria

The inclusion criteria were as follows: (1) study population should be the patients diagnosed with liver diseases and (2) galectin expression or level was detected in patients with liver diseases. The exclusion criteria were as follows: (1) duplicate papers; (2) reviews, meta-analyses, or case reports; (3) notes, conferences, corrections, editorials, comments, or letters; (4) experimental or animal studies; and (5) studies which were lacking of detailed data regarding galectin expression or level.

### Data Extraction

We extracted the following data from each study, including first author, publication year, country, study design, enrollment period, sample size, subtypes of the galectins, and methods to detect the galectins. As for the studies regarding the clinicopathological features and the outcomes of HCC, we specifically extracted the data as follows: galectin expression and its grouping; clinicopathological features including tumor size, tumor-node-metastasis (TNM) stage, differentiation grade, and vascular invasion; and outcomes, which include overall survival (OS), disease-free survival (DFS), and relapse-free survival (RFS). As for the studies regarding the risk of liver diseases, we specifically extracted the data regarding the type of liver diseases, the Child–Pugh class, and the level of serum galectins.

As for the survival data, we directly extracted or indirectly estimated the hazard ratio (HR) and 95% CI. If a study did not give the HR and 95% CI, but only reported the Kaplan–Meier curves, we would employ the Engauge Digitizer 4.1 software (Linux, Mac OSX, and Windows Slashdot Media, CA, USA) to extract the survival rate at the different time points from the Kaplan–Meier curves and then utilize Tierney's table (12) to estimate its correlative HR with 95% CI.

### Study Quality Assessment

Quality of the case–control and cohort studies were evaluated by the Newcastle–Ottawa Scale (NOS), which included the three parts (i.e., selection, comparability, and outcomes) and eight questions (13). The highest NOS score was nine points. High quality was considered if the NOS score was more than six points.

### Statistical Analysis

The Stata version 12.0 (Stata Corporation, College Station, Texas, USA) was employed for the statistical analysis. Only a random effects model was implemented. HRs, odds ratios (ORs), and mean differences (MDs) with 95% CIs were pooled. A two-sided *p* < 0.05 was considered as statistically significant. If the data were expressed as median with range, mean with SD would be estimated (14). Heterogeneity was evaluated by the *I*^2^ statistics and the Cochran's *Q* test. *I*^2^ > 50% or *p* < 0.1 was considered as a statistically significant heterogeneity. Sensitivity analysis was performed after omitting one study at a time in order to check the consistency to estimate the overall effect. Publication bias was assessed by Egger's test (15) and *p* < 0.1 was considered to imply a significant publication bias.

## Results

### Study Selection and Characteristics

**Figure 1:**
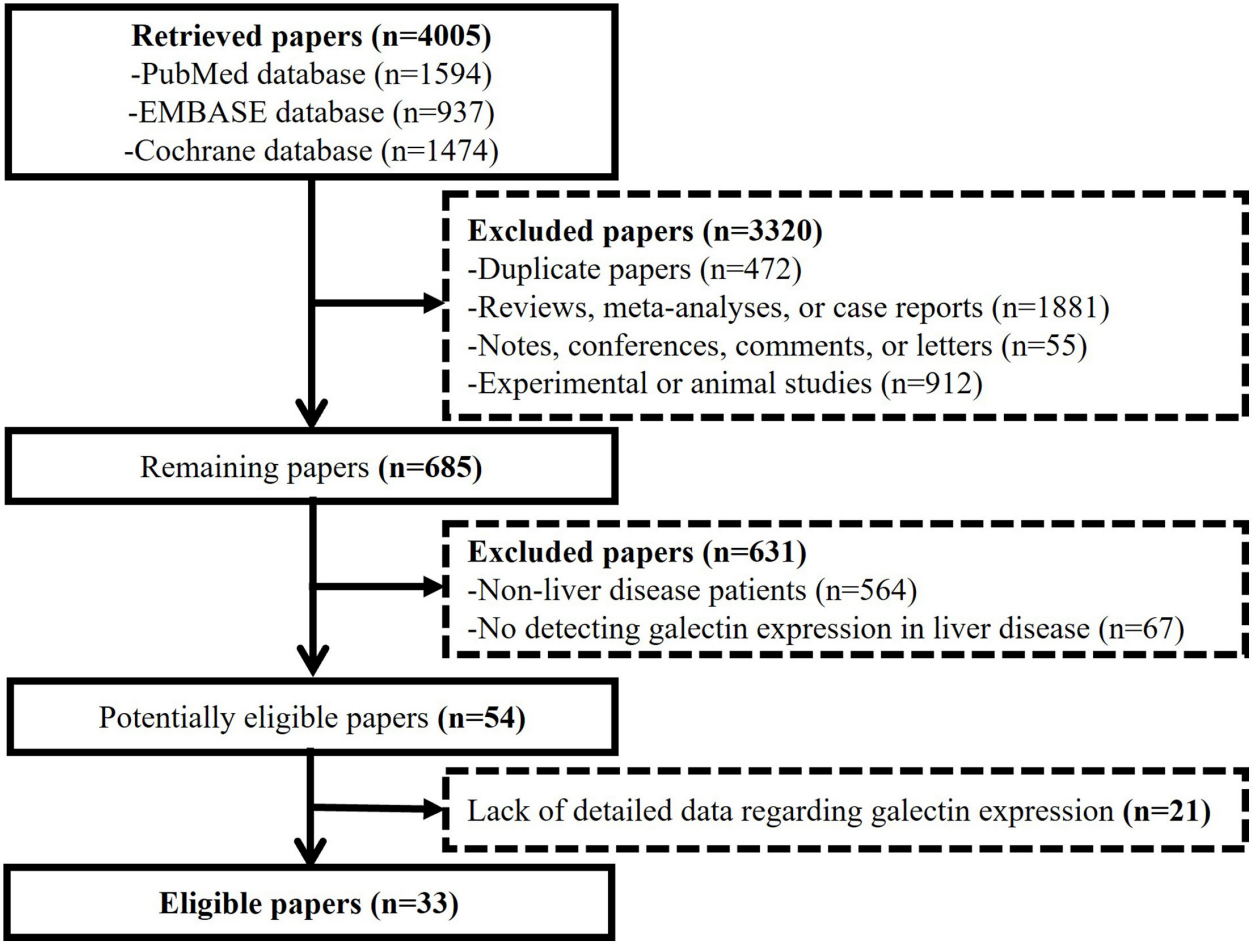
Flow diagram for the selection of the papers.

Among the 4,005 papers initially retrieved, 33 papers were eligible (Figure 1). They were published from 2008 to 2020 (16–48). Members of the galectins evaluated included galectin-1,-3,-4, and -9. The sample size ranged from 10 to 386; 25 studies came from Asia (16–20, 22, 25–39, 42, 44, 46, 47), six studies came from Europe (21, 40, 41, 43, 45, 48), and two studies came from Oceania (23, 24); five studies were published as the abstracts (27, 28, 35, 37, 42) and 28 studies were published as the full texts (16–26, 29–34, 36, 38–41, 43–48); and 29 studies were of high quality (16–26, 29, 30, 32–39, 41–48), but four studies were of low quality (27, 28, 31, 40).

### Meta-Analyses Regarding the Galectins With Prognosis and Clinicopathological Features of the Hepatocellular Carcinoma

Seventeen studies involving 19 cohorts and 3,120 patients focused on the relationship of the galectins expressed in the tissues with prognosis and clinicopathological features of HCC (16–32) (Table 1). Among them, five study cohorts focused on galectin-1 (17–21), seven study cohorts focused on galectin-3 (22, 25–29, 31), one study cohort focused on galectin-4 (32), and six study cohorts focused on galectin-9 (16, 23, 24, 26, 30). Results of the meta-analyses are shown in Table 2.

**Table 1:** Characteristics of the included studies regarding the galectins with the prognosis and clinicopathological features of HCC in the tissues.

**References**	**Country**	**Type of** **publication**	**Enrollment period**	**No. total pts**.	**Galectin subtypes**	**No. high expression**	**Pathological stage**	**IHC positive**	**Outcomes**	**Clinicopathologic features**	**HR with 95% CI**	**NOS score**
Matsuda et al. (25)	Japan	Full text	1994–2003	52	Galectin-3	34	TNMII-IV	NA	OS	Report	Survival curve	8
Spano et al. (21)	Italy	Full text	1988–2007	197	Galectin-1	44	TNMII-IV	Score>2	NA	Report	NA	7
Fang et al. (31)	China	Full text	2001–2007	46	Galectin-3	36	TNMI-III	Score>2	NA	Report	NA	5
Zhang et al. (16)	China	Full text	1995–2005	200	Galectin-9	113	TNMI-IV	Score>2	OS	Report	Survival curve	7
Wu et al. (20)	China	Full text	Up to 2011 3/15	386	Galectin-1	189	TNMI-IV	NA	OS, RFS	Report	Report	6
Gu et al. (30)	China	Full text	2006.06–2008.08	147	Galectin-9	68	TNMI-IV	NA	OS, RFS	Report	Survival curve	8
Jiang et al. (29)	China	Full text	2001–2004	165	Galectin-3	135	NA	2+ or 3+	OS	Report	Report	7
Cai et al. (32)	China	Full text	2005–2011	201	Galectin-4	89	TNMI-IV	2+ or 3+	OS, RFS	Report	Report	7
Kong et al. (26)	China	Abstract	NA	110	Galectin-3	52	NA	NA	OS	NA	Report	5
Kong et al. (28)	China	Full text	2008.10–2012.09	197	Galectin-9	106	TNMI-III	Score>100	OS	Report	Report	8
				197	Galectin-3	77					Report	
Yeh et al. (19)	China	Full text	2007–2012	91	Galectin-1	52	NA	2+ or 3+	OS	NA	Survival curve	8
Zhang et al. (17)	China	Full text	NA	209	Galectin-1	128	TNMI-IV	ICH>20%	OS	NA	Survival curve	6
You et al. (18)	China	Full text	2009–2011	162	Galectin-1	105	TNMI-IV	2+ or 3+	OS	Report	Report	7
Kong et al. (17)	China	Abstract	NA	247	Galectin-3	116	NA	NA	OS	NA	Report	5
Sideras et al. (24)	Netherlands	Full text	2001.06–2014.06	60	Galectin-9	46	TNMI-III	2+ or 3+	OS	NA	Survival curve	7
				94	Galectin-9	73					Report	
Sideras et al. (23)	Netherlands	Full text	2007.01–2013.03	81	Galectin-9	65	TNMI-III	NA	OS	NA	Report	6
Song et al. (22)	China	Full text	2005–2008	278	Galectin-3	135	TNMI-III	2+ or 3+	OS	Report	Report	7

*HCC, hepatocellular carcinoma; Pts., number of patients; NA, not available; IHC, immunohistochemistry; NOS, Newcastle–Ottawa Scale; OS, overall survival; RFS, relapse-free survival; HR, hazard ratio*.

**Table 2:** Galectins with the prognosis and clinicopathological features of HCC: results of the meta-analyses.

				**Heterogeneity**	
**Groups**	**No. studies**	**Pooled proportion using** **random-effects mode**	***P*** **-value**	***I*** ^**2**^	***P*** **-value**
**OS**					
Galectin-1	4	HR = 1.94 (95% CI = 1.61–2.34)	** <0.001**	0.0%	0.739
Galectin-3	6	HR = 3.29 (95% CI = 1.62–6.68)	**0.001**	90.0%	**0.008**
Galectin-4	1	HR = 0.53 (95% CI = 0.36–0.79)	**0.002**	–	–
Galectin-9	6	HR = 0.56 (95% CI = 0.44–0.71)	** <0.001**	3.7%	0.393
**RFS**					
Galectin-1	1	HR = 1.62 (95% CI = 1.26–2.08)	** <0.001**	–	–
Galectin-4	1	HR = 0.65 (95% CI = 0.47–0.89)	**0.008**	–	–
Galectin-9	1	HR = 0.46 (95% CI = 0.26–0.82)	**0.009**	–	–
**Tumor size**					
Galectin-1	2	OR = 1.59 (95% CI = 0.74–3.41)	0.238	75.8%	**0.042**
Galectin-3	4	OR = 1.69 (95% CI = 1.01–2.84)	**0.046**	48.8%	0.119
Galectin-4	1	OR = 0.43 (95% CI = 0.20–0.91)	**0.027**	–	–
Galectin-9	3	OR = 0.98 (95% CI = 0.70–1.39)	0.924	0.0%	0.394
**TNM stage**					
Galectin-1	2	OR = 2.53 (95% CI = 1.31–4.87)	**0.006**	41.9%	0.189
Galectin-3	4	OR = 2.06 (95% CI = 0.82–5.16)	0.122	66.6%	**0.030**
Galectin-4	1	OR = 0.49 (95% CI = 0.28–0.86)	**0.013**	–	–
Galectin-9	1	OR = 0.44 (95% CI = 0.20–0.98)	**0.044**	–	–
**Differentiation grade**					
Galectin-1	3	OR = 0.96 (95% CI = 0.70–1.32)	0.795	0.0%	0.830
Galectin-3	4	OR = 2.13 (95% CI = 0.97–4.69)	0.061	65.6%	**0.033**
Galectin-4	1	OR = 0.35 (95% CI = 0.16–0.78)	**0.010**	–	–
Galectin-9	3	OR = 0.70 (95% CI = 0.34–1.47)	0.348	70.2%	**0.035**
**Vascular invasion**					
Galectin-1	2	OR = 1.74 (95% CI = 1.18–2.58)	**0.005**	0.0%	0.679
Galectin-3	2	OR = 2.98 (95% CI = 1.58–5.60)	**0.001**	0.0%	0.421
Galectin-4	1	OR = 0.36 (95% CI = 0.19–0.72)	**0.003**	–	–
Galectin-9	2	OR = 0.60 (95% CI = 0.37–0.97)	**0.037**	2.8%	0.311

*HCC, hepatocellular carcinoma; OS, overall survival; RFS, relapse-free survival; HR, hazard ratio; OR, odds ratio. The values in bold is defined as being statistically significant*.

#### Overall Survival

The relationship between the galectins and OS was explored in 17 study cohorts (16–20, 22–30, 32).

High galectin-1 expression was significantly correlated with worse OS in the patients with HCC (HR = 1.94, 95% CI = 1.61–2.34, *p* < 0.001) without significant heterogeneity (*I*^2^ = 0.0%, *p* = 0.739).

High galectin-3 expression was significantly correlated with worse OS in the patients with HCC (HR = 3.29, 95% CI = 1.62–6.68, *p* = 0.001) with a significant heterogeneity (*I*^2^ = 90.00%, *p* = 0.008). Sensitivity analysis illustrated that the study by Song et al. (22) displayed an apparent influence on the overall result of the meta-analysis (Supplementary Figure 1). After the exclusion of this study, the pooled HR was similar (HR = 2.51, 95% CI = 1.51–4.16, *p* < 0.001), but with a mild reduction in heterogeneity (*I*^2^ = 71.10%, *p* = 0.008).

High galectin-4 expression was significantly correlated with better OS in the patients with HCC (HR = 0.53, 95% CI = 0.36–0.79, *p* = 0.002).

High galectin-9 expression was significantly correlated with better OS in the patients with HCC (HR = 0.56, 95% CI = 0.44–0.71, *p* < 0.001) without significant heterogeneity (*I*^2^ = 3.7%, *p* = 0.393).

#### Relapse-Free Survival

The relationship between the galectins and RFS was explored in three study cohorts (20, 30, 32).

High galectin-1 expression was significantly correlated with worse RFS in the patients with HCC (HR = 1.62, 95% CI = 1.26–2.08, *p* < 0.001).

High galectin-4 (HR = 0.65, 95% CI = 0.47–0.89, *p* = 0.008) and galectin-9 (HR = 0.46, 95% CI = 0.26–0.82, *p* = 0.009) expression were significantly correlated with better RFS in the patients with HCC.

#### Tumor Size

The relationship between the galectins and tumor size was explored in 10 study cohorts (16, 18, 20, 22, 25, 26, 29, 30, 32).

High galectin-1 expression was not significantly associated with tumor size (OR = 1.59, 95% CI = 0.74–3.41, *p* = 0.238) with a significant heterogeneity (*I*^2^ = 75.8%, *p* = 0.042).

High galectin-3 expression was significantly associated with bigger tumor size (OR = 1.69, 95% CI = 1.01–2.84, *p* = 0.046) without significant heterogeneity (*I*^2^ = 48.8%, *p* = 0.119).

High galectin-4 expression was significantly associated with smaller tumor size (OR = 0.43, 95% CI = 0.2–0.91, *p* = 0.027); by contrary, high galectin-9 expression was not significantly associated with tumor size (OR = 0.98, 95% CI = 0.7–1.39, *p* = 0.924) without significant heterogeneity (*I*^2^ = 0.0%, *p* = 0.394).

#### Tumor-Node-Metastasis Stage

The relationship between the galectins and TNM stage was explored in eight study cohorts (18, 21, 22, 25, 26, 31, 32).

High galectin-1 expression was significantly associated with advanced TNM stage (OR = 2.53, 95% CI = 1.31–4.87, *p* = 0.006) without significant heterogeneity (*I*^2^ = 41.9%, *p* = 0.189).

High galectin-3 expression was not significantly associated with TNM stage (OR = 2.06, 95% CI = 0.82–5.16, *p* = 0.122) with a significant heterogeneity (*I*^2^ = 66.6%, *p* = 0.030). Sensitivity analysis illustrated that the study by Kong et al. (26) displayed an apparent influence on the overall result of the meta-analysis (Supplementary Figure 2). After the exclusion of this study, the pooled OR was similar (OR = 2.90, 95% CI = 1.84–4.56, *p* = 0.044), but the heterogeneity was statistically insignificant (*I*^2^ = 0.0%, *p* = 0.731).

High galectin-4 (OR = 0.49, 95% CI = 0.28–0.86, *p* = 0.013) and galectin-9 (OR = 0.44, 95% CI = 0.20–0.98, *p* = 0.044) expression were significantly associated with early TNM stage.

#### Differentiation Grade

The relationship between the galectins and tumor differentiation grade was explored in 11 study cohorts (16, 18, 20–22, 26, 29–32).

High galectin-1 expression was not significantly associated with differentiation grade (OR = 0.96, 95% CI = 0.7–1.32, *p* = 0.795) without significant heterogeneity (*I*^2^ = 0.0%, *p* = 0.830).

High galectin-3 expression was not significantly associated with differentiation grade (OR = 2.13, 95% CI = 0.97–4.69, *p* = 0.061) with a significant heterogeneity (*I*^2^ = 65.6%, *p* = 0.033). Sensitivity analysis demonstrated that the study by Fang et al. (31) displayed an apparent influence on the overall result of the meta-analysis (Supplementary Figure 3). After the exclusion of this study, the pooled OR was similar (OR = 1.65, 95% CI = 1.01–2.69, *p* = 0.044), but the heterogeneity was statistically insignificant (*I*^2^ = 18.5%, *p* = 0.293).

High galectin-4 expression was significantly associated with well-differentiation grade (OR = 0.35, 95% CI = 0.16–0.78, *p* = 0.010).

High galectin-9 expression was not significantly associated with tumor differentiation grade (OR = 0.70, 95% CI = 0.34–1.47, *p* = 0.348) with a significant heterogeneity (*I*^2^ = 70.2%, *p* = 0.035). Sensitivity analysis illustrated that the study by Gu et al. (30) displayed an apparent influence on the overall result of the meta-analysis (Supplementary Figure 4). After the exclusion of this study, the pooled OR was similar (OR = 0.51, 95% CI = 0.28–0.95, *p* = 0.034), but the heterogeneity was statistically insignificant (*I*^2^ = 35.0%, *p* = 0.215).

#### Vascular Invasion

The relationship between the galectins and vascular invasion was explored in seven study cohorts (16, 20–22, 25, 30, 32).

High galectin-1 expression was significantly associated with positive vascular invasion (OR = 1.74, 95% CI = 1.18–2.58, *p* = 0.005) without significant heterogeneity (*I*^2^ = 0.0%, *p* = 0.679).

High galectin-3 expression was significantly associated with positive vascular invasion (OR = 2.98, 95% CI = 1.58–5.60, *p* = 0.001) without significant heterogeneity (*I*^2^ = 0.0%, *p* = 0.421).

High galectin-4 expression was significantly associated with negative vascular invasion (OR = 0.36, 95% CI = 0.19–0.72, *p* = 0.003).

High galectin-9 expression was significantly associated with negative vascular invasion (OR = 0.60, 95% CI = 0.37–0.97, *p* = 0.037) without significant heterogeneity (*I*^2^ = 2.8%, *p* = 0.311).

### Meta-Analyses Regarding the Galectins With the Risk of Different Liver Diseases

About 18 studies involving 24 cohorts and 1,048 patients focused on the relationship between the serum galectin levels and the risk of different liver diseases (25, 30, 33–48) (Table 3). Among them, 16 studies focused on galectin-3 (25, 33–38, 40–48), and two studies focused on galectin-9 (30, 39). Results of the meta-analyses are shown in Table 4.

**Table 3:** Characteristics of the included studies regarding the galectins with the risk of different liver diseases.

**References**	**Country**	**Study design**	**Type of publication**	**Enrollment period**	**Target population**	**No. total pts**.	**Child-Pugh A/B/C**	**Galectin subtypes**	**Measure-ment**	**NOS score**
Matsuda et al. (25)	Japan	Retrospective case control	Full text	2005.06–2008.02	HCC	51	38/12/1	Galectin-3	ELISA	8
					LC	16	12/2/2			
					Hepatitis	23	23/0/0			
Honsawek et al. (47)	Thailand	Retrospective case control	Full text	NA	Biliary Atresia	58	NA	Galectin-3	ELISA	6
Yilmaz et al. (34)	Turkey	Retrospective case control	Full text	NA	NAFLD	71	NA	Galectin-3	ELISA	7
Giebultowicz et al. (43)	Poland	Retrospective case control	Full text	NA	HCC	10	NA	Galectin-3	ELISA	6
Gu et al. (30)	China	Prospective cohort	Full text	2006.06–2008.08	HCC	31	NA	Galectin-9	ELISA	8
Kamada et al. (46)	Japan	Retrospective cohort	Full text	NA	NASH	127	NA	Galectin-3	ELISA	6
Yang et al. (35)	China	Prospective cohort	Abstract	NA	Liver Failure	55	NA	Galectin-3	ELISA	6
Zheng et al. (33)	China	Retrospective case control	Full text	2010.01–2011.12	Liver Failure	55	NA	Galectin-3	ELISA	8
Eisa et al. (41)	Egypt	Retrospective case control	Full text	2012.03–2012.09	HCC	50	21/18/11	Galectin-3	ELISA	8
Ulu et al. (36)	Turkey	Retrospective case control	Full text	2009–2011	HCC	19	NA	Galectin-3	ELISA	6
					LC	22				
Akyuz et al. (42)	Turkey	Retrospective case control	Abstract	NA	HCC	60	37/21/2	Galectin-3	ELISA	6
Gudowska et al. (40)	Poland	Retrospective case control	Full text	NA	LC	57	NA	Galectin-3	CMIA	5
Uluca et al. (44)	Turkey	Retrospective case control	Full text	NA	CAHB	32	NA	Galectin-3	ELISA	6
					IHB	30				
Abbas et al. (48)	Egypt	Retrospective case control	Full text	2015.08–2015.11	LC with ascites	25	0/8/17	Galectin-3	ELISA	7
					LC without ascites	26	18/8/0			
Tekin et al. (37)	Turkey	Prospective case control	Abstract	NA	CAHB	56	NA	Galectin-3	ELISA	6
					IHB	57				
Lukic et al. (45)	Bosnia and Herzegovina	Retrospective case control	Full text	NA	Hepatitis C	20	NA	Galectin-3	ELISA	8
Moon et al. (38)	Korea	Retrospective case control	Full text	2016.10–2017.02	LC	28	NA	Galectin-3	ELISA	7
Matsuoka et al. (39)	Japan	Retrospective case control	Full text	NA	AIH	77	NA	Galectin-9	ELISA	6

*Pts., number of patients; NA, not available; NOS, Newcastle–Ottawa Scale; AIH, autoimmune hepatitis; LC, liver cirrhosis; CAHB, chronic active hepatitis B; IHB, inactive hepatitis B; HCC, hepatocellular carcinoma; NAFLD, nonalcoholic fatty liver disease; LF, liver failure*.

**Table 4:** Galectins with the risk of different liver diseases: results of the meta-analyses.

				**Heterogeneity**	
**Groups**	**No. studies**	**Pooled proportion using** **random-effects mode**	***P*** **-value**	***I*** ^**2**^	***P*** **-value**
**HCC**					
Galectin-3	5	MD = 2.71 (95% CI = 1.56–3.85)	** <0.001**	86.9%	** <0.001**
Galectin-9	1	MD = 3.74 (95% CI = 2.57–4.91)	** <0.001**	–	–
**Liver failure**					
Galectin-3	2	MD = 0.44 (95% CI = 0.23–0.66)	** <0.001**	97.8%	** <0.001**
**Liver cirrhosis**					
Galectin-3	6	MD = 1.83 (95% CI = 1.15–2.51)	** <0.001**	98.7%	** <0.001**
**Chronic liver diseases**					
Galectin-3 in CAHB	2	MD = 18.95 (95% CI = 10.91–27.00)	** <0.001**	73.1%	**0.054**
Galectin-3 in IHB	2	MD = 1.29 (95% CI = −1.40–3.97)	0.347	58.9%	0.119
Galectin-3 in NASH	1	MD = 0.48 (95% CI = −0.77–1.73)	0.452	–	–
Galectin-3 in Hepatitis	1	MD = 0.37 (95% CI = −0.65–1.39)	0.479	–	–
Galectin-3 in Hepatitis C	1	MD = −0.27 (95% CI = −0.34 to−0.20)	** <0.001**	–	–
Galectin-3 in NAFLD	1	MD = 0.10 (95% CI = −0.30–0.50)	0.485	–	–
Galectin-3 in BA	1	MD = 1.30 (95% CI = 1.11–1.49)	** <0.001**	–	–
Galectin-9 in AIH	1	MD = 8.80 (95% CI = 7.61–9.99)	** <0.001**	–	–

*HCC, hepatocellular carcinoma; MD, mean difference; NA, not available; CAHB, chronic active hepatitis B; IHB, inactive hepatitis B; NASH, nonalcoholic steatohepatitis; NAFLD, nonalcoholic fatty liver disease; BA, biliary atresia; AIH, autoimmune hepatitis. The value in bold is defined as being statistically significant*.

#### Hepatocellular Carcinoma

The relationship between the galectins and the risk of HCC was explored in six study cohorts (25, 30, 36, 41–43). Among them, five study cohorts selected the healthy volunteers as the control subjects, and one study cohort selected the patients with chronic hepatitis as the control subjects.

Serum galectin-3 level was significantly higher in the patients with HCC compared to the healthy volunteers or the patients with chronic hepatitis (MD = 2.71, 95% CI = 1.56–3.85, *p* < 0.001) with a significant heterogeneity (*I*^2^ = 86.9%, *p* < 0.001). Sensitivity analysis illustrated that the study by Akyuz et al. (42) displayed an apparent influence on the overall result of the meta-analysis (Supplementary Figure 5). After the exclusion of this study, the pooled MD was similar (MD = 2.28, 95% CI = 2.07–2.50, *p* < 0.001), but the heterogeneity was statistically insignificant (*I*^2^ = 0.6%, *p* = 0.389).

Serum galectin-9 level was significantly higher in the patients with HCC compared to the healthy volunteers (MD = 3.74, 95% CI = 2.57–4.91, *p* < 0.001).

#### Liver Failure

The relationship between galectin-3 and the risk of liver failure was explored in two study cohorts, both of which selected the healthy volunteers as the control subjects (33, 35).

Serum galectin-3 level was significantly higher in the patients with liver failure compared to the healthy volunteers (MD = 0.44, 95% CI = 0.23–0.66, *p* < 0.001) with a significant heterogeneity (*I*^2^ = 97.8%, *p* < 0.001).

#### Liver Cirrhosis

The relationship between galectin-3 and the risk of liver cirrhosis was explored in six study cohorts, all of which selected healthy volunteers as the control subjects (25, 36, 38, 40, 48).

Serum galectin-3 level was significantly higher in the patients with liver cirrhosis compared to the healthy volunteers (MD = 1.83, 95% CI = 1.15–2.51, *p* < 0.001) with a significant heterogeneity (*I*^2^ = 98.3%, *p* < 0.001). Sensitivity analysis did not find any source of heterogeneity.

#### Other Chronic Liver Diseases

The relationship between the galectins and the risk of other chronic liver diseases, including inactive hepatitis B, chronic active hepatitis B, non-alcoholic steatohepatitis, hepatitis C, autoimmune hepatitis, non-alcoholic fatty liver disease, and biliary atresia, was explored in 10 study cohorts. All of them selected healthy volunteers as the control subjects (25, 34, 37, 39, 44–47).

**Figure 2:**
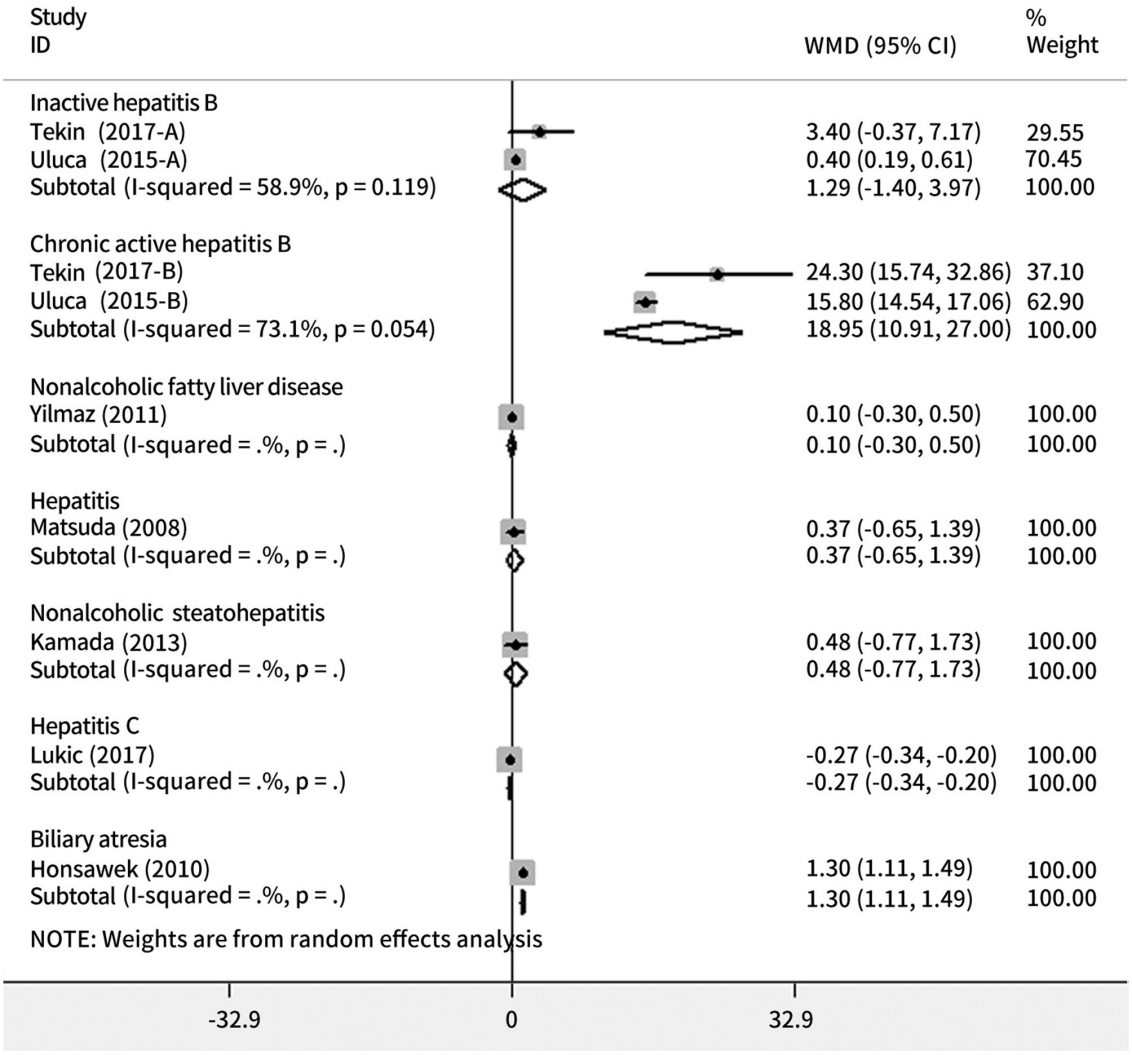
Forest plots showing the association between the risk of chronic liver diseases and galectin-3.

In comparison to the healthy volunteers, serum galectin-3 level was significantly higher in chronic active hepatitis B (MD = 18.95, 95% CI = 10.91–27.00, *p* < 0.001) and biliary atresia (MD = 1.30, 95% CI = 1.11–1.49, *p* < 0.001), but not inactive hepatitis B (MD = 1.29, 95% CI = 1.40–3.97, *p* = 0.347), non-alcoholic steatohepatitis (MD = 0.48, 95% CI = 0.77–1.73, *p* = 0.452), hepatitis (MD = 0.37, 95% CI = 0.65–1.39, *p* = 0.479), or non-alcoholic fatty liver disease (MD = 0.10, 95% CI = 0.30–0.50, *p* = 0.485); on the contrary, serum galectin-3 level was significantly lower in hepatitis C (MD = 0.27, 95% CI = 0.34–0.20, *p* < 0.001) (Figure 2).

Serum galectin-9 level was significantly higher in the patients with autoimmune hepatitis compared to the healthy volunteers (MD = 8.80, 95% CI = 7.61–9.99, *p* < 0.001).

### Publication Bias

Publication bias is reported in Supplementary Table 1.

## Discussion

Until now, 11 subtypes of galectins family have been identified in humans, of which galectin-1,-3, and -9 are the most commonly studied in various diseases (49). According to the current systematic analyses, the role of galectin-1,-3,-4, and -9 was studied in patients with liver diseases.

Patients with HCC have a 5-year survival rate of <12% (50). Therefore, it is vital to identify the biomarkers to predict the prognosis of HCC (51). This study found that the higher serum galectin-3 and -9 levels were associated with an increased risk of HCC and high galectin-1 and -3 and low galectin-4 and -9 expression were significantly associated with worse OS and positive vascular invasion in HCC. Indeed, experimental studies have also suggested the potential mechanisms of galectin-1,-3, and -9 expression in the development and progression of HCC. First, galectin-1 can induce the epithelial–mesenchymal transition (EMT), which is a major process during the progression of cancer in the HCC cells of humans (52). Galectin-1 inhibitor combined with sorafenib can further decrease the tumor size (53). Second, galectin-3 can inhibit the tumor-reactive T cells and promote tumor growth in the mice receiving the tumor-reactive CD8^+^ T cells (54). Silencing of galectin-3 can significantly reduce the mRNA and protein levels of urokinase-type plasminogen activator receptor (uPAR) and downstream signaling transduction pathway of uPARs in the HCC cells by inhibiting the MEK/ERK signaling pathway, further influencing the proliferation, migration, and invasion of the HCC cells (55). Third, galectin-9 can inhibit the growth of the HCC cell lines by inducing cell apoptosis (56). Galectin-9 also increases the number of Tim-3^+^ dendritic cells and CD8^+^ T cells and enhances antitumor immunity through the interaction of galectin-9 with Tim-3 (57). By comparison, blockade of the Tim-3/galectin-9 signaling pathway importantly increases the functionality of tumor-infiltrating Tim-3^+^ T cells and is negatively associated with the survival of patients with HCC (58).

Another major finding of this study was that higher serum galectin-3 level was associated with an increased risk of liver failure, liver cirrhosis, and chronic active hepatitis B. Other evidence was also in favor of the importance of galectin-3 in these liver diseases. First, if the patients with acute-on-chronic liver failure related to hepatitis B had galectin-3 methylated promoter, they would have shorter survival time, higher 3-month mortality, and higher model for end-stage liver disease (MELD) score (59). Second, galectin-3 modulates the phagocytosis-induced hepatic stellate cell activation and liver fibrosis *in vivo* (60). Galectin-3 level is significantly higher in the Child–Pugh class C and positively correlates with the MELD score, suggesting the association of galectin-3 level with hepatic decompensation (61). By comparison, the galectin-3 inhibitor can reduce the hepatic venous pressure gradient in patients with esophageal varices (62). Third, galectin-3 deficiency can lead to a significant reduction in the incidence of concanavalin A-induced hepatitis in mice by inhibiting inflammation (63).

This study did not find any significant association of serum galectin-3 level with inactive hepatitis B, non-alcoholic steatohepatitis, or non-alcoholic fatty liver disease. This illustrated that the impact of galectin-3 level on chronic liver diseases might be dependent upon the severity and stage of liver damage (40). Indeed, the evidence regarding the role of galectin-3 in non-alcoholic fatty liver disease and non-alcoholic steatohepatitis is also controversial. Some studies have shown that galectin-3 deficiency in male mice can spontaneously develop non-alcoholic fatty liver disease and more severe hepatic injury (64, 65). In contrast, other studies have reported that galectin-3 ablation protected the mice from the diet-induced non-alcoholic steatohepatitis (66).

There were several limitations in this study. First, this meta-analysis contained a relatively small number of studies, which might lead to insufficient statistical power. Second, the cutoff values of high galectin expression were heterogeneous among the studies. Third, HR values were not directly reported in the six included studies, where their survival data were extracted from the Kaplan–Meier curves by the Engauge Digitizer 4.1 software. Fourth, most of the included studies were from Asia. Our findings are not a global representation.

**Figure 3:**
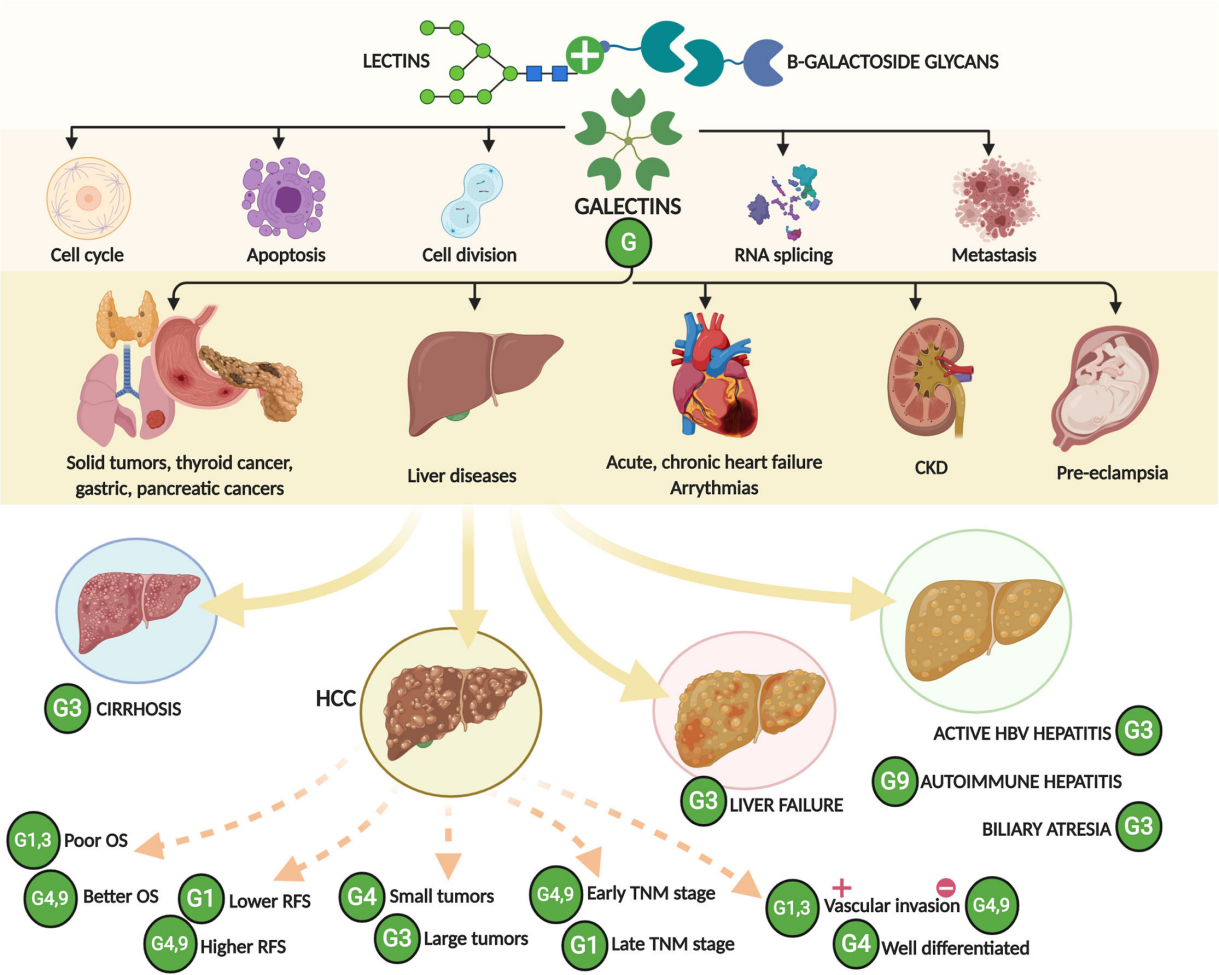
A schematic drawing showing the major findings of this study. CKD, chronic kidney disease; OS, overall survival; RFS, relapse-free survival; HCC, hepatocellular carcinoma; TNM, tumor-node-metastasis.

In conclusion, based on this systematic review and meta-analysis, both high galectin-1 and -3 and low galectin-4 and -9 expression in the tissues were significantly related to worse prognosis and positive vascular invasion in patients with HCC and serum galectin-3 level was associated with the risk of HCC, liver failure, liver cirrhosis, and chronic active hepatitis B (Figure 3). Further studies are needed to explore the role of galectins as a potential therapeutic target and biomarker for liver diseases.

## Data Availability Statement

The original contributions presented in the study are included in the article/Supplementary Material, further inquiries can be directed to the corresponding author/s.

## Author Contributions

XQ contributed to the conceptualization, supervision, and project administration. YA, SX, YL, XX, and XQ contributed to the methodology, formal analysis, data curation, and writing the original draft. YA, SX, YL, XX, CP, JC, NM-S, XG, and XQ contributed to the validation, writing, review, and editing. All authors contributed to the article and approved the submitted version.

## Conflict of Interest

The authors declare that the research was conducted in the absence of any commercial or financial relationships that could be construed as a potential conflict of interest.

## Publisher's Note

All claims expressed in this article are solely those of the authors and do not necessarily represent those of their affiliated organizations, or those of the publisher, the editors and the reviewers. Any product that may be evaluated in this article, or claim that may be made by its manufacturer, is not guaranteed or endorsed by the publisher.

## Supplementary Material

Supplementary Figure 1 Sensitivity analysis of galectin-3 expression with overall survival (OS) in HCC.

Supplementary Figure 2 Sensitivity analysis of galectin-3 expression with TNM stage in HCC.

Supplementary Figure 3 Sensitivity analysis of galectin-3 expression with the differentiation grade in HCC.

Supplementary Figure 4 Sensitivity analysis of galectin-9 expression with the differentiation grade in HCC.

Supplementary Figure 5 Sensitivity analysis of serum galectin-3 level with the risk of HCC.

Supplementary Table 1 Publication bias.
