# Supplementary figures and images for: Role of Galectins in the Liver Diseases: A Systematic Review and Meta-Analysis
Source: Front Med (Lausanne). 2021 Oct 27;8:744518. doi: 10.3389/fmed.2021.744518 (PMC8578830; doi:10.3389/fmed.2021.744518)

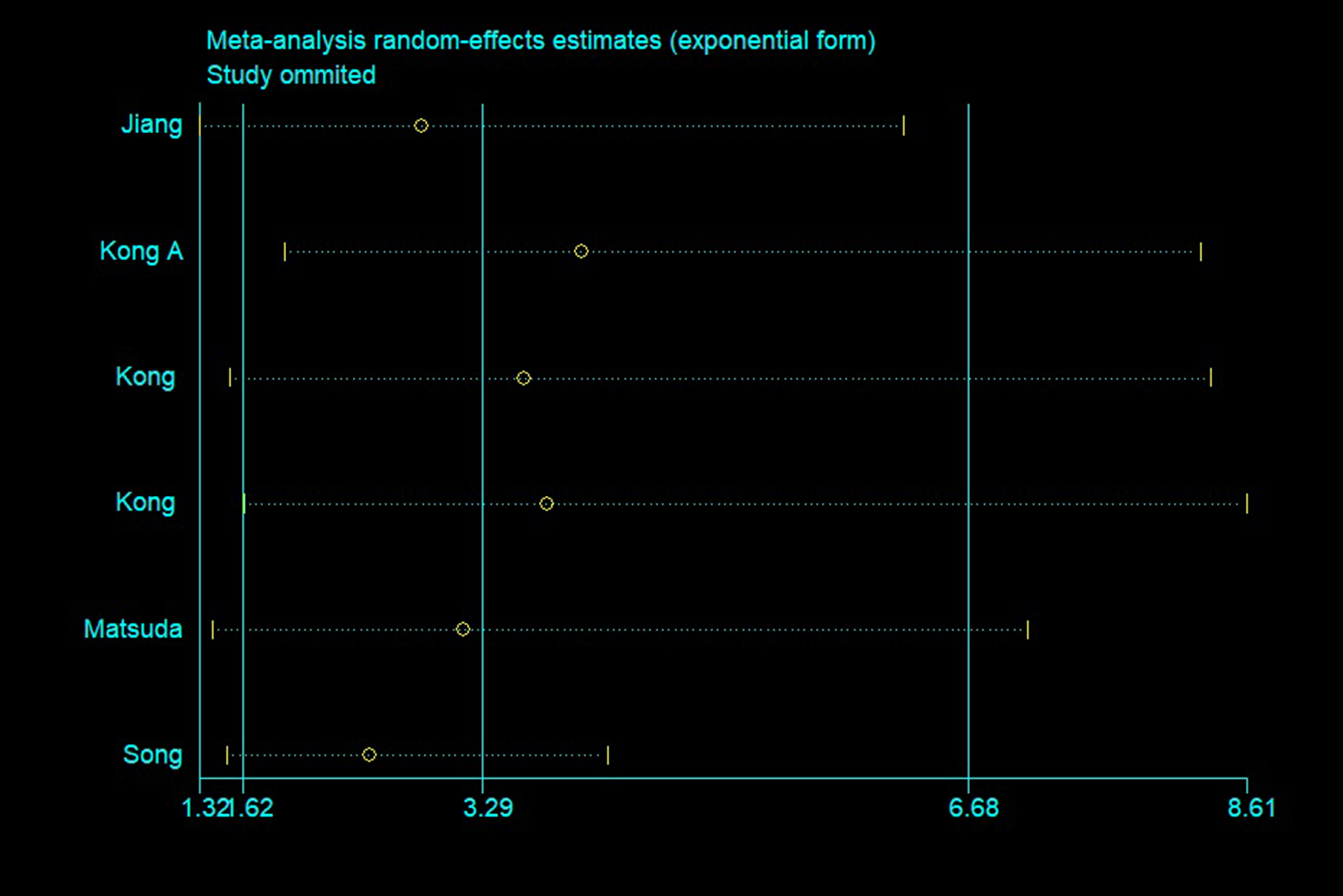

Supplement: Supplementary Figure 1 — Sensitivity analysis of galectin-3 expression with overall survival (OS) in HCC. [file Image_1.JPEG]

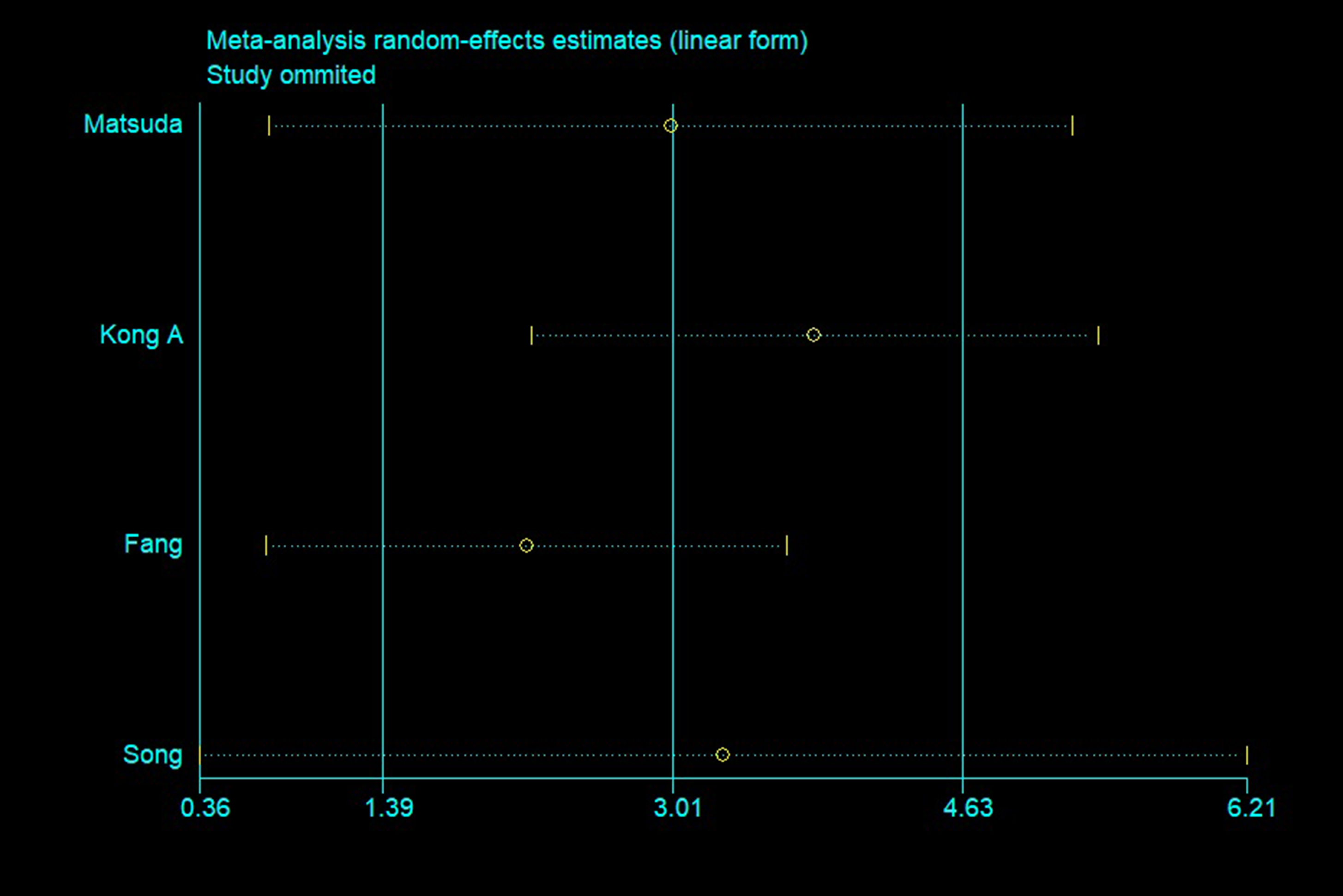

Supplement: Supplementary Figure 2 — Sensitivity analysis of galectin-3 expression with TNM stage in HCC. [file Image_2.JPEG]

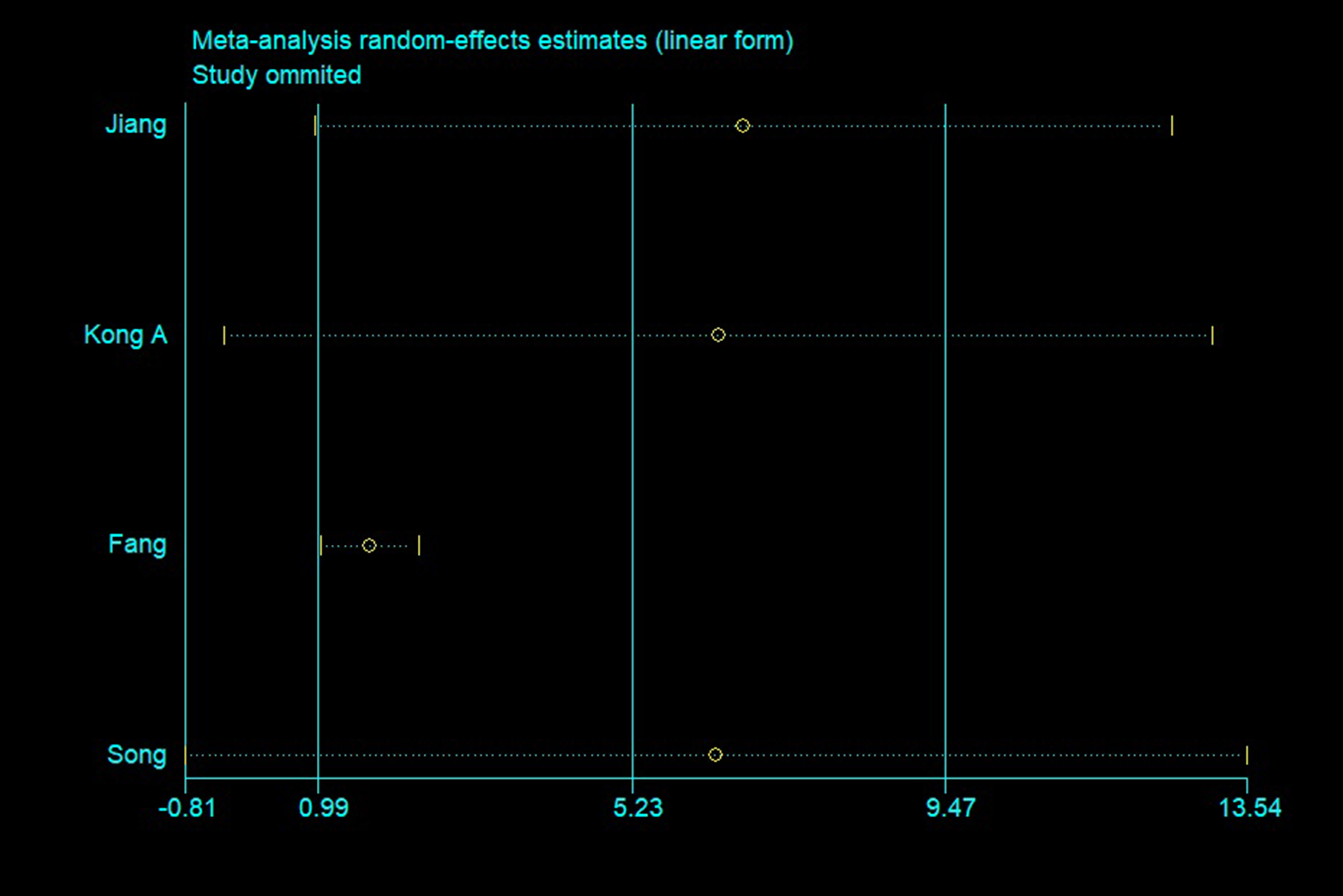

Supplement: Supplementary Figure 3 — Sensitivity analysis of galectin-3 expression with the differentiation grade in HCC. [file Image_3.JPEG]

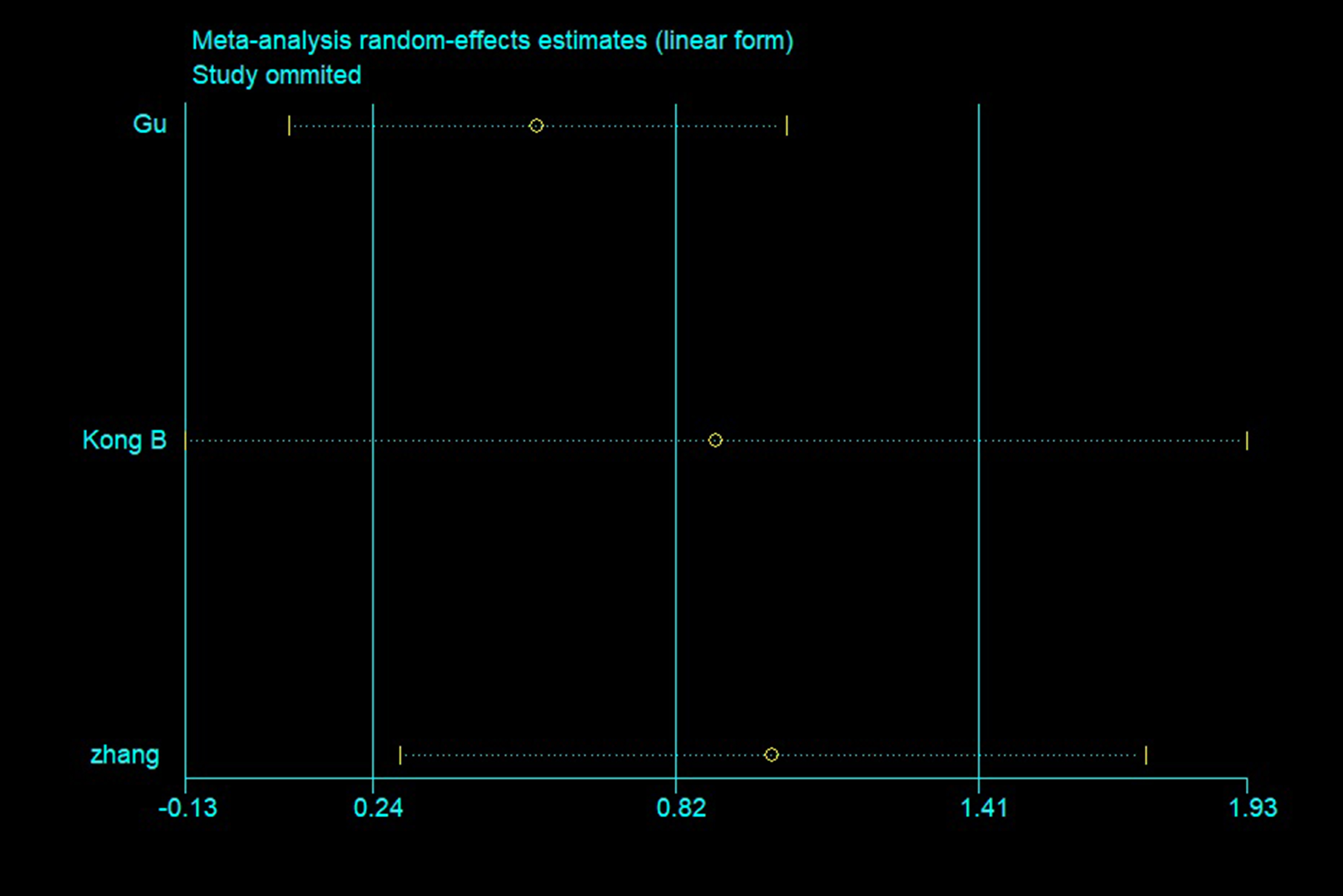

Supplement: Supplementary Figure 4 — Sensitivity analysis of galectin-9 expression with the differentiation grade in HCC. [file Image_4.JPEG]

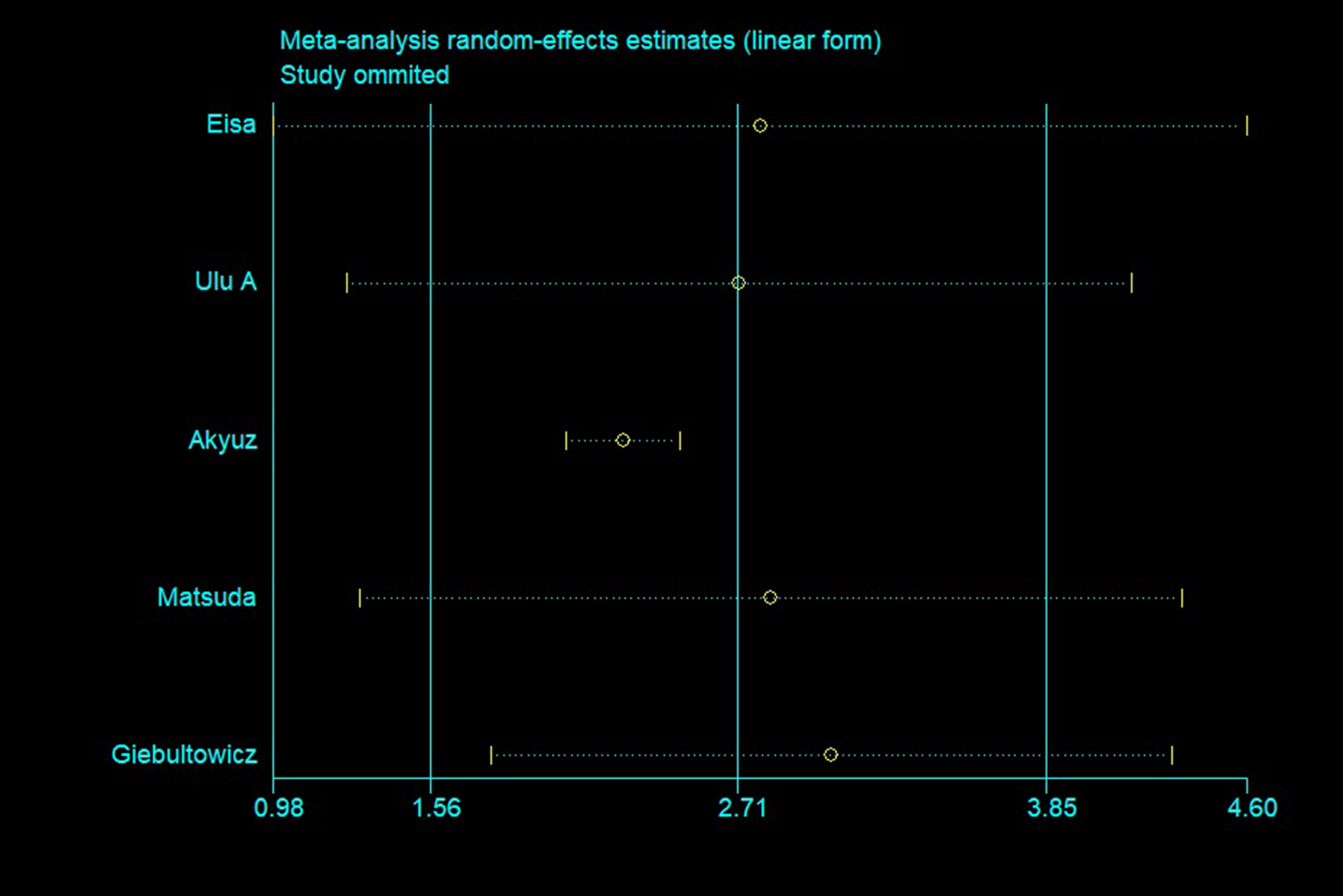

Supplement: Supplementary Figure 5 — Sensitivity analysis of serum galectin-3 level with the risk of HCC. [file Image_5.JPEG]
