# Supplementary material for: Role of Galectins in the Liver Diseases: A Systematic Review and Meta-Analysis
Source: Front Med (Lausanne). 2021 Oct 27;8:744518. doi: 10.3389/fmed.2021.744518 (PMC8578830; doi:10.3389/fmed.2021.744518)
Supplement: Supplementary Table 1 — Publication bias. [file Table_1.DOCX]

Supplementary Table 1. Publication bias

| **Variable** | **r** | **Coefficient** | **Standard error.** | **t** | **P** | **95% Confidence interval** | |
| --- | --- | --- | --- | --- | --- | --- | --- |
| ***Overall survival in HCC*** | | | | | | | |
| Galectin-1 | bias | 0.5138747 | 1.625041 | 0.32 | 0.782 | -6.478113 | 7.505862 |
| Galectin-3 | bias | -0.6409989 | 5.007242 | -0.13 | 0.904 | -14.54333 | 13.26133 |
| Galectin-9 | bias | -1.70728 | 1.040168 | -1.64 | 0.176 | -4.595251 | 1.18069 |
| ***Tumor size in HCC*** | | | | | | | |
| Galectin-3 | bias | 0.2060505 | 2.956533 | 0.07 | 0.946 | -6.611726 | 7.023827 |
| Galectin-9 | bias | -2.161637 | 8.069257 | -0.27 | 0.833 | -104.6913 | 100.368 |
| ***TNM stage in HCC*** | | | | | | | |
| Galectin-3 | bias | 1.629612 | 3.899338 | 0.42 | 0.717 | -15.14789 | 18.40711 |
| ***Differentiation grade in HCC*** | | | | | | | |
| Galectin-1 | bias | 1.345793 | 0.6354257 | 2.12 | 0.281 | -6.728056 | 9.419642 |
| Galectin-3 | bias | 18.90255 | 10.95617 | 1.73 | 0.227 | -28.23803 | 66.04313 |
| Galectin-9 | bias | -2.208142 | 8.868909 | -0.25 | 0.845 | -114.8983 | 110.482 |
| ***Risk of galectin-3 with liver diseases*** | | | | | | | |
| HCC | bias | 1.069045 | 1.925583 | 0.56 | 0.617 | -5.059019 | 7.197109 |
| Liver cirrhosis | bias | 7.658903 | 3.043686 | 2.52 | **0.066** | -.7917233 | 16.10953 |
